# Hospitals and the Honours List

**Published:** 1920-04-17

**Authors:** 


### The Hospital

The Workers' Newspaper of Administrative Medicine and Institutional Life, Administration, National Insurance and Health.

No. 1767, Vol. LXVIII. SATURDAY, APRIL 17, 1920. PRICE TWOPENCE

### HOSPITALS AND THE HONOURS LIST.

It was necessary to wait until the voluminous list of civilian war honours had been virtually completed before a judgment could be formed on the degree of "official gratitude" for the work of the voluntary hospital in the war. As the matter stands at present, only five hospital secretaries have been decorated, and of these five, four only in connection with the war. We single the hospital secretary for mention because he is the administrative, or at least the executive, head, and on his office, therefore, rests the main burden, though in the last resort the responsibility for the policy which the secretary pursues lies with the committee and the chairman. Of the five secretaries who have been decorated Mr. P. J. Michelli received the C.M.G. in 1906. The Order indicated the recognition of the Colonial Office for services rendered to the London School of Tropical Medicine associated with the Seamen's Hospital, Greenwich. In the succeeding fourteen years but four other decorations have been bestowed upon his secretarial colleagues. Of the five decorations bestowed four have been given to London secretaries and one to a provincial secretary. Mr. G. Q. Roberts, of St. Thomas's Hospital, received the C.B.E., an Order which implies recognition for his work in the war, and for the honourable record which preceded it. Then, in the most recent list, Mr. J. C. Buchanan received the same decoration, which in turn suggests appreciation of his work at the Metropolitan Hospital during the war. It coincided with his departure from that institution and auspiciously marked his new appointment at the Cancer Hospital. At the same time we were delighted to see the name of Mr. H. Wade Deacon, who in his double capacity as chairman of the Lancashire Local War Pensions Committee and of the council of the British Hospitals Association, has certainly earned official gratitude and recognition. Mr. Buchanan is also honorary secretary to the British Hospitals Association; but it is probable that what we may call his institutional work and Mr. Deacon's in regard to pensions, were officially the prevailing claims. The C.B.E. conferred on Mr. E. W. Morris, House Governor of the London Hospital, and the O.B.E. upon Mr. Arthur Morley, of the Royal National Orthopædic Hospital, are also well deserved.

Well, grateful as we are for the recognition which has befallen the men already mentioned, it must be admitted that the amount of recognition accorded to hospital men is disappointingly small. To suppose that distinctions are invidious is a democratic fallacy, especially when distinctions are deserved. No hospital secretary would care to claim a more distinguished record than that of Mr. Frank Hazell, of the Manchester Royal Infirmary, or than that of Mr. Roden Orde, of Newcastle. It is strictly true that their names are conspicuous by their absence from the Honours List; and, though we lament the absence, we may congratulate them on a distinction which is too genuine to be dimmed by want of artificial light. We are all in their debt, and are proud to acknowledge it. It is, however, with the profession that we have especially to do.

It is still relatively new, insufficiently recognised; and it needed the war to set the seal upon its importance. The honours which it has gained mark a step in advance, and it rests with the profession to make its claim to public recognition as unchallengable in practice as it is already undisputed in theory. To secure this two things have to be done. The first is a further revision of salaries, so that the best brains may be attracted to it. This revision must include a proper system of pensions, for the plums of the profession are still few and far between, and there is only limited scope for promotion. Secondly, the most careful selection must be made in the matter of appointments, so that at least the best material at present available may be encouraged. We do not want the profession to boast of a few choice spirits at the head, if the rank and file are undistinguished, and contain many unfit by character and education for promotion. That has been the weakness hitherto. But it would be unfair to dissociate this weakness from the meagre rewards open to hospital talent. The two defects should be borne equally in mind. The correction of each will help to counteract the other; and as the counteraction proceeds, administrative medicine will be recognised to be among the great professions, and we shall cease to lament its absence from the Honours List.

